# Visual perception of order-disorder transition

**DOI:** 10.3389/fpsyg.2015.00734

**Published:** 2015-06-10

**Authors:** Mikhail Katkov, Hila Harris, Dov Sagi

**Affiliations:** Department of Neurobiology, Weizmann Institute of ScienceRehovot, Israel

**Keywords:** phase transition, symmetry, order parameter, visual texture, perceptual organization

## Abstract

Our experience with the natural world, as composed of ordered entities, implies that perception captures relationships between image parts. For instance, regularities in the visual scene are rapidly identified by our visual system. Defining the regularities that govern perception is a basic, unresolved issue in neuroscience. Mathematically, perfect regularities are represented by symmetry (perfect order). The transition from ordered configurations to completely random ones has been extensively studied in statistical physics, where the amount of order is characterized by a symmetry-specific order parameter. Here we applied tools from statistical physics to study order detection in humans. Different sets of visual textures, parameterized by the thermodynamic temperature in the Boltzmann distribution, were designed. We investigated how much order is required in a visual texture for it to be discriminated from random noise. The performance of human observers was compared to Ideal and Order observers (based on the order parameter). The results indicated a high consistency in performance across human observers, much below that of the Ideal observer, but well-approximated by the Order observer. Overall, we provide a novel quantitative paradigm to address order perception. Our findings, based on this paradigm, suggest that the statistical physics formalism of order captures regularities to which the human visual system is sensitive. An additional analysis revealed that some order perception properties are captured by traditional texture discrimination models according to which discrimination is based on integrated energy within maps of oriented linear filters.

## 1. Introduction

The notion of the world as being ordered dates back to the earliest philosophical accounts. For example, the ancient Greeks expressed this notion in the word “Cosmos,” whose original meaning was “ordered world.” Our visual system perceives the external world as being well-organized. Previous works related to perceptual organization were based on concepts rooted in information theory, Gestalt psychology, and physiology (Attneave, 1954; Barlow, 1961; Julesz, 1965; Julesz et al., 1978; Rubenstein and Sagi, 1990; Kubovy, 1994; Wagemans, 1997; Simoncelli and Olshausen, 2001; Landy and Oruç, 2002; Treder, 2010; Wagemans et al., 2012; Giannouli, 2013; van der Helm, in press). Nevertheless, the principles of perceptual organization are still largely unknown. Here we considered this issue from a new perspective, by examining whether statistical physics formalism can be used to describe human perception.

According to the Gestalt school of psychology, several basic laws govern image segmentation and pattern formation, such as the law of proximity, similarity, and symmetry, which define symmetries within the input image. Moreover, when studying the symmetry law, mirror symmetry is mostly considered experimentally (Treder, 2010), whereas different symmetries are involved in dot lattices (see relevant discussion in Kubovy, 1994; Kubovy and Wagemans, 1995). Therefore, the results obtained in studies of all the three Gestalt laws, mentioned above, are likely to depend on symmetry perception. An analogy can be drawn to theoretical physics, where each conserved physical measure has a corresponding symmetry, as described by Noether's first theorem (Noether, 1918), for instance, the time invariance associated with the energy conservation law.

The link established between Gestalt psychology and information theory (Attneave, 1954) allows one to consider the Gestalt laws in the framework of efficient coding. It has been suggested that the human sensory system is sensitive to statistical regularities in the external world. Previous research, aimed at identifying the underlying statistics represented by the visual system, frequently utilized visual textures (Julesz, 1965; Julesz et al., 1978; Fogel and Sagi, 1989; Rubenstein and Sagi, 1990; Chubb et al., 1994; Kingdom et al., 2001; Landy and Oruç, 2002; Victor and Conte, 2005; Balas, 2006; Maddess et al., 2007; Morgan et al., 2008; Freeman et al., 2013; Westrick and Landy, 2013). However, previous works mostly focused on simple statistics that are typically limited to low-order moments (Geisler, 2008). Therefore, a higher order formal account of the visual texture is required to fully address perceptual organization. Visual textures are useful stimuli for studying the principles of perceptual organization for three main reasons: (1) they can be constructed based on a formal model, (2) they cover a broad range of structures, from highly repetitive patterns with strong regularities, to completely independent local intensities, such as 1/f noise, or white noise images, and (3) visual textures involve integrating many elements over a large spatial range.

In statistical mechanics, order is tightly linked to symmetry. More specifically, a physical system is considered to be ordered if it is symmetric, that is, it is invariant with respect to a set of transformations. In contrast, a disordered system lacks symmetry. In condensed matter physics, an ordered state is observed at a range of low temperatures (an ordered phase), whereas a disordered state is observed at a range of high temperatures (a disordered phase), with a transition between them (i.e., a phase transition) at a particular temperature. Moreover, at this temperature the symmetry of the physical system changes (Landau, 1937).

One of the notable models used to describe the phase transition is the Ising model, which was originally used to explain ferromagnetism (Brush, 1967). In short, it assumes that discrete variables, representing magnetic dipoles, are placed in a two-dimensional grid. Each variable can be in one of two states (+1, −1), reflecting up and down local magnetization, respectively. The adjacent dipoles interact, and the preferred configuration is one in which they have the same magnetization—this state corresponds to low potential energy. Thermal fluctuations counteract this tendency. Therefore, at low temperatures all dipoles are arranged in the same state (an ordered state), whereas at very high temperatures the states are almost independent (a disordered state) with transition occurring at intermediate temperatures. Importantly, in this framework the amount of order is objectively defined as the mean conformance of local configurations to global symmetry (Sethna, 2006). Technically, one need to define a symmetry-specific local measure at each location—an order field—having high values when local configuration is consistent with global symmetry, and having low values when it is not. The mean value of an order field represents the amount of order in a given system. Visual textures can be created to conform to this model by assuming a two-state variable placed on a square grid, with the variable corresponding to some visual feature such as color or luminance, or even a displacement of textural elements in dot lattices.

Here we propose a novel framework for studying the order sensitivity of the human visual system by applying tools from statistical mechanics. Specifically, we first designed interaction rules assuming three-state variables and the corresponding visual textures, where each state represents a specific amplitude of a Gaussian blob. We next simulated thermal equilibrium configurations for the designed textures at different temperatures. Human observers were able to discriminate between the generated images (of varying levels of order) from noise images. This framework allows one to compare the sensitivity of the human visual system and model observers—an Ideal observer, an Order observer, a Luminance observer, and a Channel Energy observer.

## 2. Results

Little is known regarding whether human perception has any correlates with the order of the stimuli defined using the statistical physics framework. Therefore, we performed a series of experiments. Experiment 1 was exploratory in nature, leading to Experiment 2, which was constrained and elaborated based on the results of Experiment 1. More specifically, the adaptive measurement method was used in Experiment 1 in order to find a range of parameter values where psychometric function changes. In Experiment 2, the method of constant stimuli was used in order to evaluate human performance with better precision. Additionally, in Experiment 1 the observers were asked to report “What quadrant of the display contained the most ordered image.” In Experiment 2 the observers were asked “What quadrant contained the exceptional image?” Thus, the observers were not explicitly requested to judge the presence of order. Finally, a control experiment (Experiment 3) was performed in order to check whether marginal luminance statistics was employed by human observers.

### 2.1. Design of stimuli

**Figure 1:**
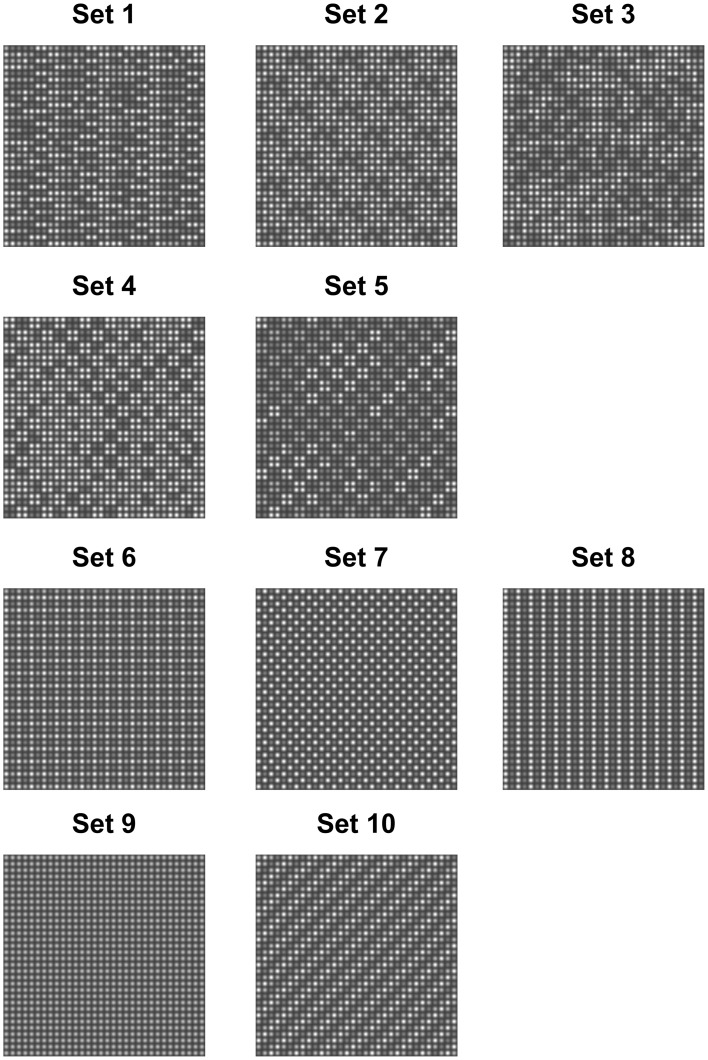
**Examples of generated textures (β = 1) used in the experiment**. Texture sets 1–5 have a different appearance across realizations, whereas Texture sets 6–10 have the same appearance (up to a shift) in different realizations.

**Figure 2:**
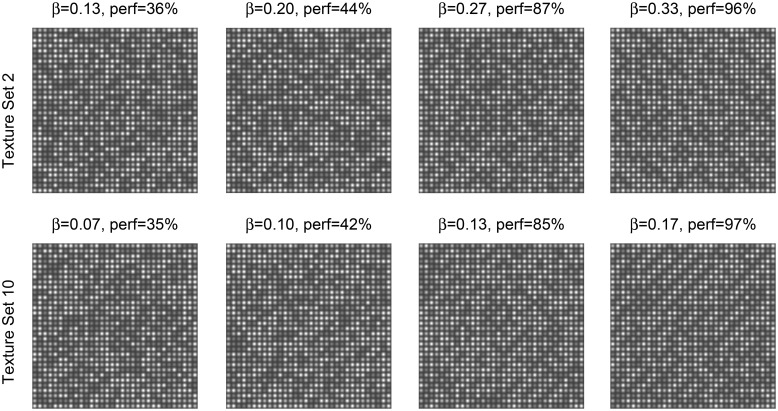
**Examples of generated textures from sets 2 and 10 for different values of β**. In the image title the value of β and the average human performance in Experiment 2 are shown.

A grid consisting of 32 × 32 Gaussian blobs was used as the visual textures. The amplitudes of the Gaussian blobs were limited to 3 different levels. Ten texture sets of visual textures were designed. Each texture set consisted of images with varying degrees of order, characterized by a single parameter $\beta =\frac{1}{T}$, which represents the inverse thermodynamic temperature in the Boltzmann distribution

$$P(I)=\frac{1}{Z}e^{-\beta U(I)},$$

where *I* represents the 32 × 32 matrix of amplitude levels and potential *U*(*I*) is set specific (see details in Methods). Parameter β controls the amount of order in the image. Images generated with small β-values represent random noise, and images with large β-values represent ordered images, with a configuration of amplitudes corresponding to a minimum value of *U*(*I*). In most of the designed sets β = 1 corresponds to periodic ordered images (Figure 1). At intermediate β-values the images have a reduced order (Figure 2).

Each display consists of one target texture and 3 distractor textures. The target texture was a synthesized image, with varying levels of order, randomly placed in one of the four quadrants of the stimulus display (see Methods). The amplitudes of distracting textures were independent uniform random variables. A spatial 4-alternative-forced-choice (4AFC) paradigm was used to measure the performance level, which is the percentage of correct discriminations between distracting images and target textures at different temperatures.

### 2.2. Sensitivity to order

**Figure 3:**
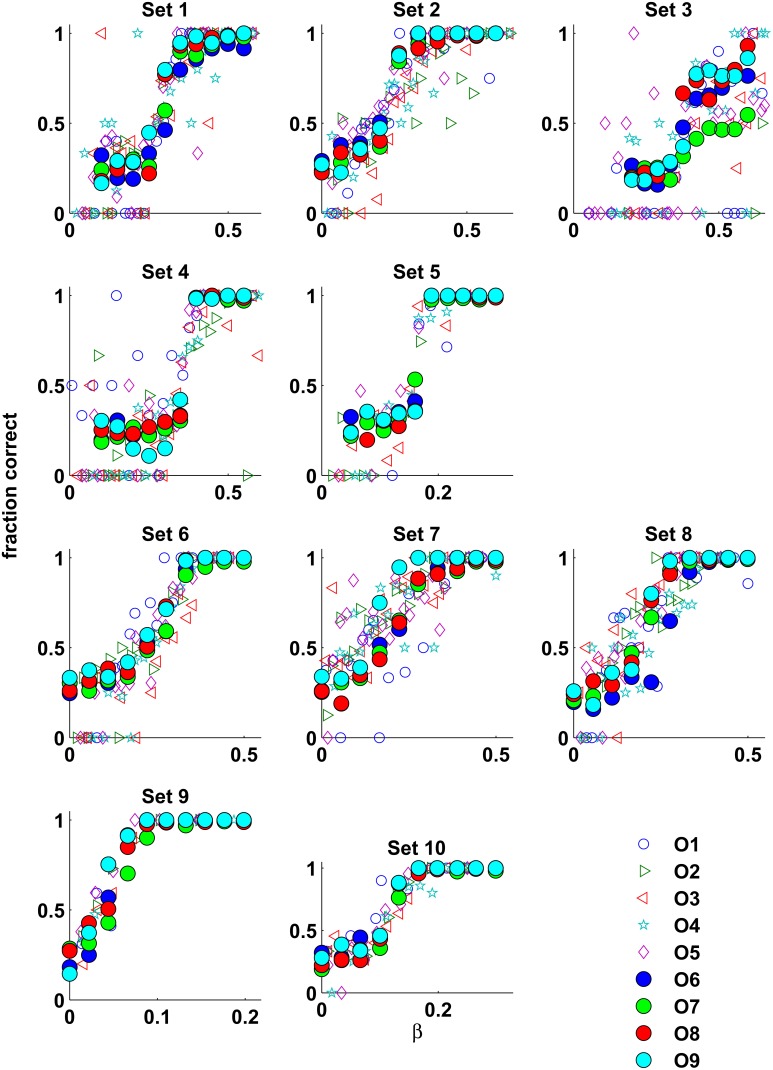
**The results of Experiment 1 (open symbols) superimposed on the results of Experiment 2 (filled symbols)**. Despite differences in experimental procedures and slight differences in the appearance of the stimulus, the performances of the human observers in both experiments are in good agreement. Moreover, in Experiment 2 there is almost a perfect match between the observers' performances.

First, we measured the performance of human observers as a function of β. Figure 3 shows the psychometric functions obtained in Experiment 1 (Observers O1–O5, open symbols) and Experiment 2 (observers O6–O9, filled symbols). For all texture sets the psychometric functions were monotonic, up to noisy estimates from the adaptive staircase method in Experiment 1. Good agreement in performance between observers was obtained in Experiment 1, and precise agreement was obtained in Experiment 2 (see Table 1).

**Table 1:** ***P*****-values of χ^2^-test (9 degrees of freedom) with the null hypothesis that the performance of a given observer is the same as the mean performance of 3 other observers**.

**Observer**	**Set 1**	**Set 2**	**Set 3**	**Set 4**	**Set 5**	**Set 6**	**Set 7**	**Set 8**	**Set 9**	**Set 10**
O6	**0.025**	0.708	0.239	0.966	0.797	0.993	0.902	**0.000**	0.667	0.399
O7	0.751	0.927	**0.000**	0.631	0.266	0.767	0.578	0.695	**0.041**	0.446
O8	0.352	0.995	**0.000**	0.947	0.604	0.918	0.867	0.205	0.815	0.909
O9	0.116	0.976	**0.036**	0.147	0.966	0.882	**0.006**	0.448	0.163	0.869

*Bold values represent P-values smaller than 5% rejecting a null hypothesis*.

The near match in performance between Experiments 1 and 2 is independent of the task, whether the observers were requested to judge which quadrant of display was more ordered, as in Experiment 1, or which quadrant was the “odd ball,” as in Experiment 2. This emphasizes the abstract and intrinsic notion of order. Furthermore, to eliminate any texture-set-specific strategy that influences performance, in Experiment 2 the textures from all sets were randomly mixed across trials. Moreover, in this experiment, in contrast to Experiment 1, the textures in the stimulus display were attached to each other to determine whether border processing plays a role in task performance. Thus, the consistent performance shown in Experiment 2 reveals the existence of a basic order perception mechanism utilized by the human visual system. Moreover, this mechanism integrates information over an extended spatial region, not only regions around the texture border.

Unlike the performance corresponding to the other texture sets, the performance corresponding to texture set 3 was never perfect, even for the lowest temperature (the largest β-value). In addition, different observers reached different performance plateaus. Nevertheless, the informative range of β, where the observer's performance changes, is consistent across all observers. Apparently, the order in this texture set is perhaps masked by the embedded noise. In particular, images from texture set 3 generated at the largest β-value (see Figure 1) can be described as a low contrast order embedded in high-contrast noise.

### 2.3. Order perception is independent of marginal luminance distribution

**Figure 4:**
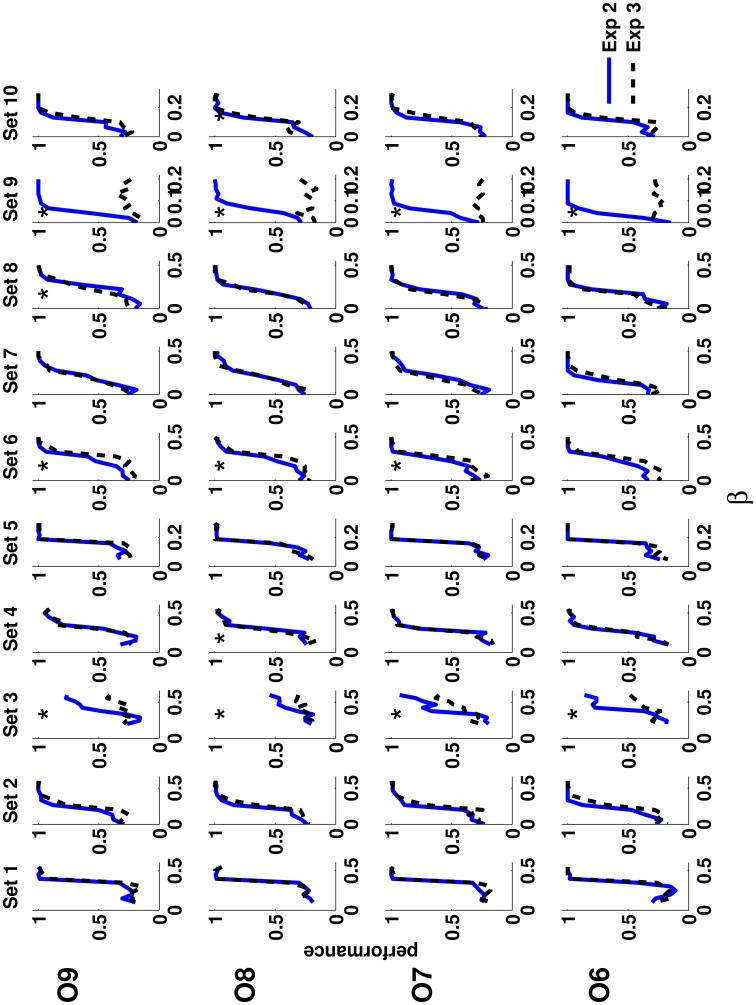
**The performance of the human observers in Experiment 2 (solid blue lines) and in Experiment 3 (dashed black lines)**. The performance is shown for each observer (rows) and for each texture set (columns) separately. In nearly all cases, except for Texture sets 3 and 9, the performances in the two experiments are almost identical. Note that the luminance distribution was identical for target and distracting quadrants in Experiment 3, whereas in Experiment 2 the amplitudes of Gaussian blobs in distracting quadrants were drawn from the uniform distribution of 3 values, and therefore, the luminance information could potentially be used in the task. A star denotes experimental conditions in which two curves are significantly different (*p*_*val*_ < 0.05) according to χ^2^ statistical test (9 degrees of freedom).

Here we tested the potential role of marginal luminance distribution in order perception. In Experiment 3, distracting images were constructed from the target image in each trial by randomly permuting the location of the Gaussian blobs. This operation leaves the luminance distribution unchanged, and destroys any geometrical relationship between the amplitudes of Gaussian blobs in the image. In all other respects, Experiments 2 and 3 were identical and were performed by the same observers. Figure 4 shows the psychometric functions for each observer (rows) and each texture set (columns) obtained in Experiments 2 and 3. There are hardly any differences between the pairs of psychometric functions (except texture sets 3 and 9), indicating that human observers did not use marginal luminance distribution in detecting order. Texture set 9, in its ordered state, is represented by a field of Gaussian blobs with uniform amplitudes. A permutation of such a target, to construct the distractors, results in the same field of Gaussian blobs with uniform amplitudes. Therefore, the target is practically indistinguishable from the distractors, and a chance–level performance across a range of temperatures is expected. Some deviations are possible around the order-disorder transition due to correlation properties near a critical point (Wilson and Kogut, 1974; Wilson, 1979). As mentioned earlier, texture set 3 contains high-contrast noise, masking low-contrast order. Thus, it carries a temperature-dependent marginal luminance distribution that seems to be used by human observers.

### 2.4. Theoretical observers

Here we compared human performance to different theoretical observers. The design of our visual textures allowed us to construct 4 different models of texture perception: (1) the Luminance observer considers only amplitude distributions, while disregarding the structural properties of a visual texture; (2) the Ideal observer has, by definition, access to all of the statistical information present in the visual texture, practically summarized in the sufficient statistics; (3) the Order observer is symmetry specific—a separate order observer based on the order parameter was constructed for each texture set (see Methods for details); (4) an observer based on the integrated energy within maps of oriented linear filters—the “Channel energy” observer (see Methods for details).

**Figure 5:**
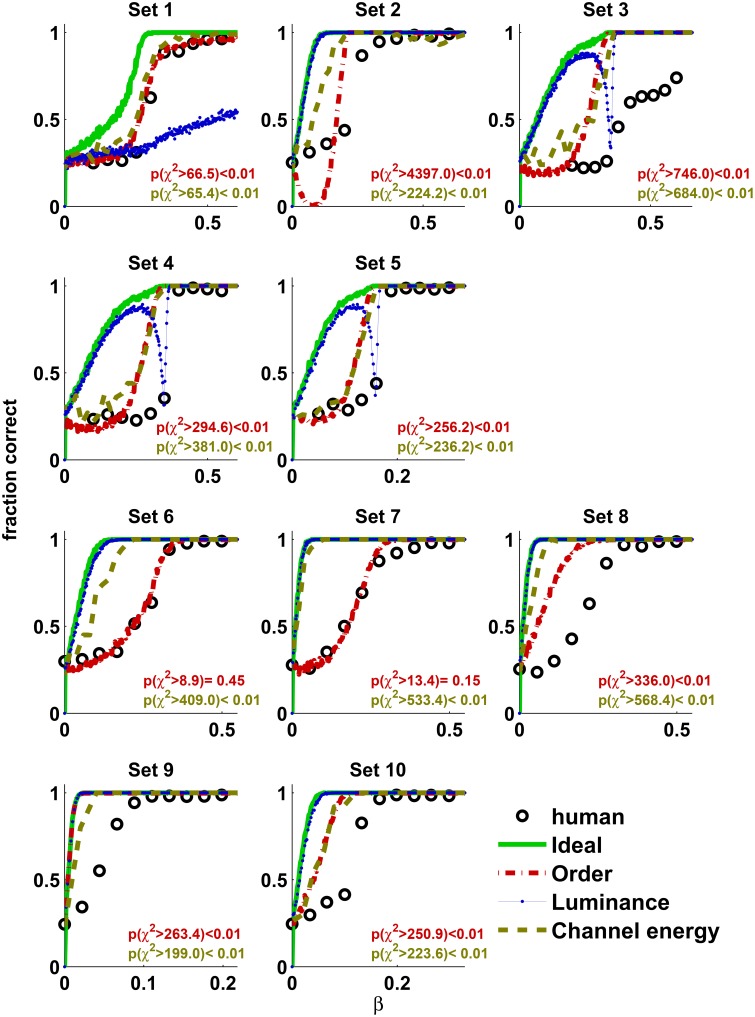
**The average performance of the human observers obtained in Experiment 2 is superimposed on the performance of the theoretical observers: Ideal, Order, Luminance, and Channel energy**. The Ideal observers were nearly perfect, whereas the human observers were at a chance level. In all cases, except one, the Luminance observer was better than the human observers. The performance of the Order observer was much closer to that of the human observers. In 3 cases (Texture sets 1, 6, and 7) there is a close match between the human and the Order observers' performances. χ^2^ statistics for Order (top) and Channel energy (bottom) observers are presented in the inset. In all cases there are 9 degrees of freedom.

**Figure 6:**
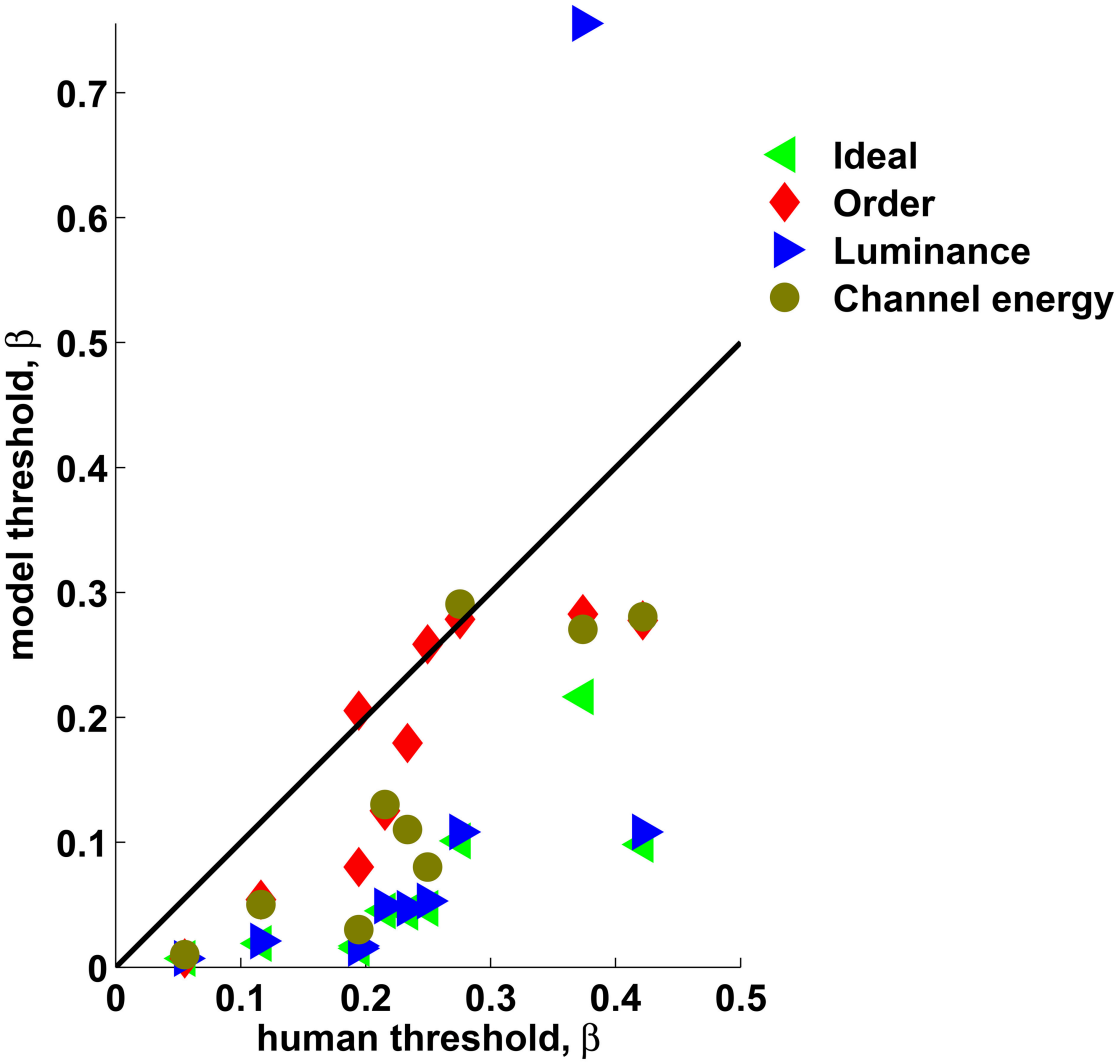
**The theoretical observers' thresholds vs. the human observers' thresholds**. The black line represents identity. All texture sets are combined. It can be seen that the Order observers' thresholds (red diamonds) approach those of the human observers and the Channel energy observers (brown circles) often approach those of the Order observers, whereas Ideal (green left triangles) and Luminance (blue right triangles) outperform the humans, except for the Luminance observer applied to Texture set 1.

Figure 5 shows the performance of Theoretical observers as a function of the thermodynamic parameter β superimposed with the mean performance of the human observers in Experiment 2. The Ideal observer was nearly perfect when human observers were still at the chance level, indicating that human observers cannot fully adapt to the statistics of the presented stimuli within the time of the experiment. The Luminance observer also outperformed the human observers in most texture sets. This result, together with the results of Experiment 3, clearly demonstrates that human observers did not utilize marginal luminance information while performing this task, even when it was available. Among the theoretical observers, the performance of the Order observer was much closer to the performance of the human observers in all texture sets. In some texture sets (1, 6, and 7) the Order observer nearly matched the human's performance. It is interesting that usually the Channel energy observer's performance approached that of the Order observer, sometimes outperforming it. This is also reflected when comparing the thresholds of the human and theoretical observers (62.5% correct discrimination, Figure 6). Note that the linear filters used for the Channel energy observer were not optimized, and, as in standard texture discrimination models, information from different linear filters was not integrated. Therefore, this observer could potentially perform better with those modifications. These results suggest that the human visual system may have mechanisms sensitive to order. This sensitivity is not governed by basic visual cues such as marginal luminance distribution or statistical cues represented by sufficient statistics. Moreover, such mechanisms may be implemented by the receptive field found in the visual cortex.

## 3. Discussion

Here we introduce a new paradigm to quantitatively study order perception in humans based on tools borrowed from statistical physics. We designed stimuli by defining the interaction rules between image parts via the potential in the Bolzmann distribution and varied the thermodynamic temperature to sample a state at thermodynamic equilibrium. At low temperatures, such a state follows the designed symmetry, but at high temperatures the image parts are completely independent. Following this route, we measured the sensitivity of the human visual system and compared it to the amount of order present in visual textures by controlling a single parameter—the thermodynamic temperature. Humans' performance in this order detection task was found to be consistent across observers, showing very small inter-observer variability. Apparently, human observers do not utilize marginal luminance distributions or a texture set-specific strategy (also, they do not rely on texture border information). Furthermore, we compared the human performance to four different theoretical observers based on different statistics of the presented images—an Ideal observer, a Luminance observer, an Order observer, and a Channel energy observer (an observer based on integrated energy within maps of oriented filters). Taken together, the performances of the Order observers were consistently closer to those of the human observers than were the Ideal and Luminance observers. Since the Ideal observer (defined by sufficient statistics) outperformed the human observers, apparently, the visual system does not construct an exact representation of the statistical properties of the presented images. Consequently, this result suggests that our observers do not adapt to the statistical properties of our synthetic stimuli, at least in the timescale of the experiment. In the framework of Signal Detection Theory, this may mean that throughout evolution the internal noise was not reduced to a level approaching the performance of an Ideal observer. In contrast, the concept of order, borrowed from statistical physics, accounts for human performance much better, without introducing a large amount of internal noise. Interestingly, with some texture sets, the performance of the channel energy observer resembled the performance of the order observer. The channel energy observer is based on integrating the energy within maps of oriented filters, which is a model frequently utilized to describe human performance in texture segregation (Landy and Graham, 2003). Together with the good across-observers agreement in performance, this suggests that the human brain is endowed with the ability to perceive order (as it is defined in statistical mechanics), which may extend beyond the visual system.

It is important to note that the Order observer was based on the order parameter—a symmetry-specific measure describing the average consistency of local patches to global symmetry in the ordered state. According to statistical physics, the order parameter reflects changes in the symmetry of the system. It has a small value in a disordered state (below a critical temperature), but it gradually grows, starting from the temperature corresponding to the phase transition. Therefore, the agreement in performance between human and Order observers indicates that the human visual system is sensitive to changes in symmetry. The texture sets used in this study do not allow us to specify explicitly the type of symmetry the visual system is sensitive to, and this is a subject for further research. Nevertheless, the agreement in performance between the Order observers and the Channel energy observer may indicate that previously identified mechanisms, such as those found in the visual cortex, underlie symmetry detection. Notably, the linear filters applied in the Channel energy observer are roughly consistent with human psychophysics as well as with the properties of simple cells in the early visual cortex (Gattass et al., 1981, 1988; Watson, 2000).

What neural mechanisms can account for order detection? In statistical physics, the value that represents the amount of order in a system is technically quantified as the mean conformance of local patches to the global symmetry (Sethna, 2006). This further suggests a mechanism in which the first stage is more local and performs non-linear computations (input-output transformations of a signal). Subsequently, those computations are summed across a visual field. Such mechanisms have been previously suggested (Rubenstein and Sagi, 1990; Landy and Graham, 2003; Westrick and Landy, 2013). The rivalry effect (bi-stable percept of two conflicting presentations, Blake and Logothetis, 2002) suggests that not only one, but many competing local symmetry detectors may be involved. Such a bi-stability is probably determined by the largest consistency of local symmetries across visual space, which changes with time due to adaptation or internal noise. An alternative mechanism is related to the properties of physical systems near the critical point that is close to a phase transition point.

At the critical temperature, when the system is at a phase transition point, from the ordered phase to the disordered phase, correlations of all lengths are present (Wilson, 1979). Near this point the system may employ autocorrelation measurements (Klein and Tyler, 1986; Ben-Av and Sagi, 1995) to detect the presence of order. How can such correlations be represented in the human brain? Several physiological findings are linked to this description. For instance, (1) cells in the early visual area, V1, respond to local features (DeValois and DeValois, 1990) that are prevalent in the disordered phase; (2) preferential activation in higher cortical areas, such as LO, is obtained in response to line drawings and objects, which can be considered as ordered because they are perceptually organized (Malach et al., 1995; Grill-Spector et al., 2001). Since the receptive field sizes increase when moving from early areas (V1) toward higher order areas (V4) (Gattass et al., 1981, 1988; Freeman and Simoncelli, 2011), one can speculate that with increasing cortical layers along the visual hierarchy, local correlations with increasing scales are initially computed. Later, local correlations are pooled to compute the amount of order present at different parts of the scene. It is predicted that more brain areas would be activated in response to observing images close to phase transition (relative to ordered or noisy images).

Interestingly, both perceptual mechanisms proposed above are similar to the ideas initially presented by Julesz (1962). The autocorrelation mechanism proposed by Klein and Tyler (1986), accounting for more than 2 luminance levels, directly extends Julesz's work. However, it did not consider any connection of autocorrelation measurements to phase transition. The other mechanism can be summarized as a first-order statistic of the output of local symmetry detectors. This resembles Julesz's notion that the visual system computes the first-order statistic of local feature detectors (Julesz, 1981). Here, instead of textons proposed in Julesz's work, we propose the involvement of non-linear local symmetry detectors.

In a recent work, natural images were decomposed into binary layers (Saremi and Sejnowski, 2013). Each layer represents different bits in a digital representation of the original image. Interestingly, it was demonstrated that layers composed of the most significant bits appeared ordered, layers composed of the least significant bits were “random,” and in between were the critical layers (corresponding to phase transition). Saremi and Sejnowski (2013) demonstrated that these critical layers contained most of the information. Assuming that the efficient coding hypothesis is true, this suggests that the representation of the critical layers plays an important role in the perception of natural images. This interpretation is supported by our findings that human perception is sensitive to order (in its physical sense—the change of symmetry).

There are three major approaches used to study perceptual organization: Gestalt psychology, efficient coding, and pattern recognition. Gestalt psychology provides a descriptive account in which several perceptual grouping laws, including the symmetry law, have been demonstrated (Kubovy, 1994; Wagemans, 1997; Treder, 2010; Giannouli, 2013; van der Helm, in press). In the framework of efficient coding, it has previously been suggested that the sensory system is tuned to the statistical properties of the environment (Attneave, 1954; Barlow, 1961; Simoncelli and Olshausen, 2001). In pattern recognition it is assumed that the human visual system consists of mechanisms sensitive to specific patterns either processed in one stage—textons (Julesz et al., 1978), or processed in two or more stages—filter-rectifier-filter models, e.g., (Rubenstein and Sagi, 1990; Landy and Oruç, 2002). It is possible that all three approaches can be unified using tools from statistical physics.

Here we propose a new experimental paradigm that naturally unifies concepts used in different fields: symmetry perception, which is rooted in Gestalt psychology (Kubovy, 1994; Wagemans, 1997; Treder, 2010; Giannouli, 2013; van der Helm, in press); texture synthesis techniques borrowed from efficient coding (Zhu et al., 1998); and the design of local filters to implement the Order observer, borrowed from physiology and psychophysics (Julesz, 1965; Julesz et al., 1978; Gattass et al., 1981, 1988; DeValois and DeValois, 1990; Rubenstein and Sagi, 1990; Landy and Oruç, 2002; Freeman and Simoncelli, 2011). Our theoretical observers were modeled using a framework of Signal Detection Theory (Green and Swets, 1966), which is a special case of a Bayesian observer with flat prior and the likelihood ratio as a decision criterion (Kersten et al., 2004).

Our findings indicate that during the early processing stages the human visual system is sensitive to order in accordance with the statistical physics definition of order. Determining what kind of order/symmetry or a combination of orders/symmetries the human visual system can represent, and what specific mechanisms are involved require further research.

## 4. Methods

### 4.1. Human search and animal research

This study was approved by the Weizmann Institute of Science Ethics Committee and the Helsinki Committee.

### 4.2. Human observers

Nine human observers with normal or corrected-to-normal vision participated in the experiment. All observers provided their informed consent, under the approved Declaration of Helsinki.

### 4.3. Stimuli

Stimuli were presented on a Philips Brilliance 109P4 CRT display. The viewing distance was 130 cm. Stimuli consisted of 1 target and 3 distracting images presented in four quadrants of the visual field, equidistantly from the fixation point (a spatial four-alternative-forced-choice paradigm). The rest of the display had a uniform luminance (26 cd/m^2^ in Experiment 1, and 14 cd/m^2^ in Experiments 2 and 3). Both the target and distracting images were composed of a 32 × 32 grid of circularly symmetrical Gaussian blobs with σ = 1.5′. The amplitudes of Gaussian blobs (*A*_*p, q*_ at location *p* and *q*) were selected across 3 different values (*a*_1_ = 0.25 *L*_max_, *a*_2_ = 0.5 *L*_max_, *a*_3_ = 0.75 *L*_max_, *L*_max_ = 100 cd/m^2^ in Experiment 1 and *a*_1_ = 26 cd/m^2^, *a*_2_ = 56 cd/m^2^, *a*_3_ = 91 cd/m^2^ in Experiments 2 and 3). Amplitude indexes for target textures were generated by sampling from a distribution defined by a set of interaction rules and a specified temperature (see Supplementary Material). Amplitude indexes for distracting images in Experiments 1 and 2 were independent random variables with uniform distribution. The distracting images in Experiment 3 were generated by randomly permuting the location of the Gaussian blobs of each target image.

Amplitude indexes were generated using the parallel Chromatic Gibbs sampling algorithm (Geman and Geman, 1984; Gonzalez et al., 2011). Sub-grids with (odd, odd), (odd, even), (even, odd), and (even, even) coordinates were updated in parallel. Each blob amplitude was updated according to the conditional distribution

$$P(A_{j,m}|A_{i,k})=\frac{1}{Z_{\left(i,k),(j,m\right)}}e^{-\beta U_{\left(i,k),(j,m\right)}(A_{i,k},A_{j,m})},$$

where *Z*_(*i,k*),(*j,m*)_ is a normalization factor, *U*_(*i,k*),(*j,m*)_ is the texture set-specific potential, and the parameter β controls the amount of order in the image. *A*_*i,k*_ ∈ {0, 1, 2}. The potential was shift invariant 3 × 3 matrix *U*_(*i,k*),(*j,m*)_ = *U*_(*i* + *p,k* + *q*),(*j* + *p,m* + *q*)_, ∀ *p,q*, and the textures had cyclic boundary conditions. The specific set of interacting pairs and the interaction potentials between them are shown in Supplementary Material.

### 4.4. Display sequence

**Figure 7:**
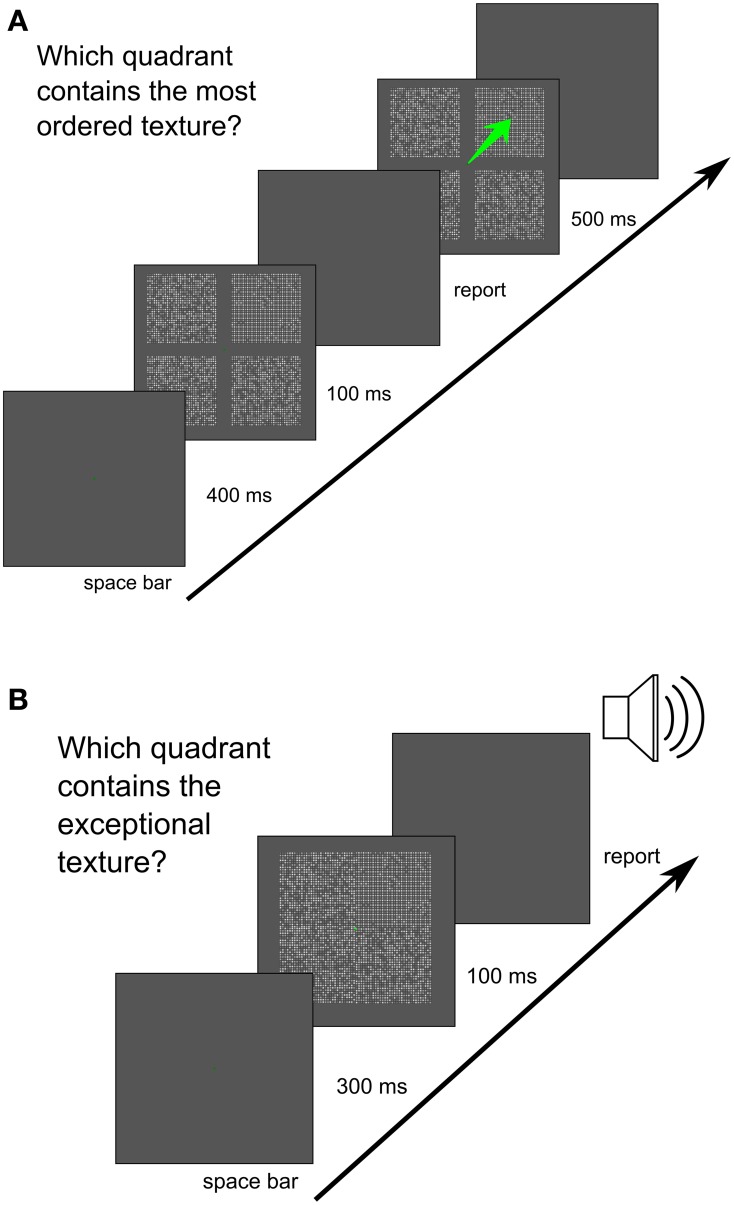
**Display sequence used in the experiment. (A)** Sequence used in Experiment 1. **(B)** Sequence used in Experiments 2 and 3.

A green fixation point on an otherwise uniform gray background was presented until the space bar was pressed by the observer. After 400 ms in Experiment 1, and 300 ms in Experiments 2 and 3, the stimulus display was shown for 8 consecutive frames at an 85 Hz refresh rate (94 ms), followed by a blank screen until the observer responded. Then, in Experiment 1 a visual feedback, repeating the stimulus display with an additional green arrow pointing at the correct response, was shown for 500 ms (observers could use saccades to glance at the correct image). In Experiments 2 and 3 auditory feedback for an incorrect response was provided. In Experiment 1, the centers of the images were 4° from the fixation point such that there was a gap of a uniform screen between textures. In experiments 2 and 3 the images (the target and three distractor textures, each 4.84° in the vertical and horizontal dimensions) were attached to each other without an overlap, forming a 64 × 64 grid of Gaussian blobs with a fixation point in the middle of the grid. The quadrant, where the target image appeared, was randomly selected at each trial. In Experiment 1, blocks of 50 trials containing textures from the same texture set were used. In Experiment 2, textures from all sets were mixed randomly; therefore, the observers could not use texture–set–specific strategies. Graphically, the sequence of frames presented is shown in Figure 7.

### 4.5. Theoretical observers

The performance of the Luminance, Order, and Ideal observers were computed in the Signal Detection Theory framework (Green and Swets, 1966). The performance of the Luminance and Ideal observers was based on the multidimensional statistics described below. The probability densities were estimated from an analysis of 1000 samples using the kernel density estimation method (Simonoff, 1998). The performance of the Order and Channel energy observers was based on a one-dimensional value (the order parameter and integrated energy in the channel, correspondingly). The performance was estimated from 1000 simulated trials (100 for the Channel energy observer) by means of computing the fraction of correct responses. For each trial it was determined whether the response having a maximum estimated likelihood (based on the estimated densities) correctly identified the target image (for details, see Supplementary Material).

#### 4.5.1. The luminance observer

The Luminance observer was defined as an observer having access to only luminance information, disregarding the geometrical structure of the image. More specifically, the statistic consists of a 3–dimensional vector. Each dimension represents the number of Gaussian blobs with amplitudes of given values (*a*_1_, *a*_2_, *a*_3_).

#### 4.5.2. The ideal observer

The Ideal observer, by definition, has access to all statistical information regarding the texture. The performance of an Ideal observer was estimated using statistics measured for each interacting pair. For each pair, a 9-dimensional statistic was measured. Each dimension represents the number of pairs of Gaussian blobs with corresponding amplitudes (see Supplementary Material). Therefore, the dimensionality of the Ideal observer statistic was 9 times the number of the interacting pairs.

#### 4.5.3. The order observer

The performance of an Order observer was based on the value of the order parameter and its variability across different samples obtained at the same temperature. The order parameter represents to what extent local patches, on average, are consistent with the overall symmetry of the image. Therefore, it is set specific. Consequently, 10 different order observers were defined. The specific details can be found in Supplementary Material.

#### 4.5.4. The channel energy observer

The performance of the Channel energy observer was based on the output of a quadrature pair of odd and even Gabor filters integrated across the quadrants of the visual field. More specifically, the output map was computed as

$$M={\left(I*G_{odd}\right)}^{2}+{\left(I*G_{even}\right)}^{2},$$

where ^*^ denotes convolution and *I* = *I(x, y)* is an image's intensity at coordinates *(x, y)*,

$$G_{even}(x,y)=e^{-\frac{x^{2}+y^{2}}{2\sigma^{2}}}\cos\left(2\pi \lambda (x\cos\theta +y\sin\theta )\right),$$

$$G_{odd}(x,y)=e^{-\frac{x^{2}+y^{2}}{2\sigma^{2}}}\sin\left(2\pi \lambda (x\cos\theta +y\sin\theta )\right),$$

σ, θ, and λ are the width, orientation, and spatial frequency of the Gabor filter. The parameters used in the simulation are presented in Table 2. The decision variable represents a sum of *M*-values over the corresponding quadrant.

**Table 2:** **Parameters of Gabor filters used in the simulation of the Channel energy observer**.

	**Set 1**	**Set 2**	**Set 3**	**Set 4**	**Set 5**	**Set 6**	**Set 7**	**Set 8**	**Set 9**	**Set 10**
σ (°)	0.085	0.085	0.085	0.123	0.123	0.085	0.085	0.085	0.085	0.085
λ (cycles/°)	6.87	6.87	6.87	6.61	6.61	6.87	6.87	6.87	6.87	6.87
θ (°)	22.5	45	45	45	45	45	45	22.5	45	45

## Author contributions

MK and DS designed the experiments. HH conducted the experiments. MK analyzed the data. MK, HH, and DS wrote the paper.

### Conflict of interest statement

The authors declare that the research was conducted in the absence of any commercial or financial relationships that could be construed as a potential conflict of interest.
