# Supplementary material for: Visual perception of order-disorder transition
Source: Front Psychol. 2015 Jun 10;6:734. doi: 10.3389/fpsyg.2015.00734 (PMC4461815; doi:10.3389/fpsyg.2015.00734)
Supplement: Supplementary file 1 [file Presentation1.PDF]

Supplementary Material: Visual perception of order-disorder transition

Mikhail Katkov [1,*], **HilaHarris** [1] **and Dov Sagi** [1]

[1] *Weizmann Institute of Science, Department of Neurobiology, Rehovot 76100, Israel*

Correspondence*:
Mikhail Katkov
Weizmann Institute of Science, Department of Neurobiology, Rehovot 76100, Israel, mikhail.katkov@gmail.com

Perceptual grouping - the state of the art

DETAILS ON EXPERIMENTAL METHODS

GENERATING RULES

The visual images used in this study consisted of a grid of 32×32 Gaussian blobs where each blob can have one of 3 possible amplitudes. Therefore, each texture can be represented by a 32×32 matrix A where each entry has one of 3 possible values – $A_{i,j} \in \{0, 1, 2\}, i = 0..31, j = 0..31$. In modeling visual textures we assumed that A is a field of random variables. For each texture set the conditional probability of one blob amplitude $A_{i,k}$ at position (i, k) as a function of another blob amplitude $A_{j,m}$ at position (j, m) had the following form:

$$P(A_{i,k}|A_{j,m}) = \frac{1}{Z_{(i,k),(j,m)}} e^{-\beta U_{(i,k),(j,m)}(A_{i,k},A_{j,m})}, \tag{1}$$

where $Z_{(i,k),(j,m)}$ is a normalization factor, $U_{(i,k),(j,m)}$ defines the set-specific interaction rules, the global parameter β defines the strength of the interactions and is analogous to the inverse of the thermodynamic temperature in the Boltzmann distribution. Therefore, only pairwise interactions were employed, and the full conditional distribution for the blob amplitude at position (i, k) is

$$P(A_{i,k}|A) = \prod_{(i,k)\neq(j,m)} P(A_{i,k}|A_{j,m}).$$

The interaction rules are set to be translation invariant and the texture was assumed to have cyclic boundary conditions.

$$U_{(i,k),(j,m)} = U_{(i+p \bmod 32, k+q \bmod 32),(j+p \bmod 32, m+q \bmod 32)},$$

for $p, q = 1..31$ (corresponding amplitudes were dropped for notation simplicity). Owing to shift invariance, it is sufficient to only specify the relative offset between the interacting blobs

$$U_{[p,q]} = U_{(i,k),(i+p \bmod 32, k+q \bmod 32)}, \forall i, k = 0..31$$

Since amplitudes were restricted to only three different levels, the interaction rule $U_{[p,q]}$ in the most general form is a 3×3 matrix for each pair $[p, q]$. A set of pairs $[p, q]$ and the corresponding interaction rules are summarized in Supplementary Table 1 (texture sets 1-4) and Supplementary Table 2 (texture sets 5-10).

Once the generating rule and β are fixed, one can generate the field A with the equilibrium state using, for example, the Gibbs sampler. The Gibbs sampler computes $P(A_{i,k}|A)$ at some location (i, k); then it assigns a new value for $A_{i,k}$ in accordance with an amplitude drawn from a computed distribution. This operation is repeated many times. Finally, under some conditions, and after many repetitions, all amplitudes have corresponding probability distributions, independent of the initial conditions. This property can be used in order to generate images with specific symmetry. At low temperatures (large β values) only configurations with the smallest energy levels are possible, whereas at high temperatures ($\beta = 0$) all configurations are equiprobable.

COMPUTING THE GENERATING RULES FROM THE SYMMETRY

For all symmetries used in this work, it is possible to construct the interaction rules. One needs to design a set of non-conflicting conditional probabilities that define a desired configuration. For example, an ordered stimulus from texture set 8 is a vertical grating where the vertical lines with amplitudes a_1 are intermixed with the vertical lines with amplitudes a_3. Therefore, generating rules reflecting this symmetry - the vertical translation by one position leaves the configuration unchanged, and the horizontal shift by one position changes a_1 to a_3 and vice versa a_3 to a_1. These rules do not contradict each other, since they operate in orthogonal directions. Therefore, to implement these rules, one needs at least two interacting pairs $[0, 1]$ (a vertical shift by one location) and $[1, 0]$ (a horizontal shift by one location) for each Gaussian blob. The interaction rule for a vertical shift is

$$U_{[0,1]} = \begin{bmatrix} 0 & \infty & \infty \\ 0 & \infty & 0 \\ \infty & \infty & 0 \end{bmatrix}.$$

Each row of this matrix represents the potential for three possible amplitudes: a_1, a_2, a_3 conditioned on the amplitude of the neighboring blob. More specifically, the first row represents the potential for the amplitudes when the neighboring blob immediately on the bottom has amplitude a_1. The second row represents potential when the neighboring blob has amplitude a_2, and so forth. The numbers in the corresponding rows are used to compute the probability of an updated amplitude in the Gibbs sampler. For instance, ∞ represents impossible states, and 0 represents possible states. When two zeros appear in the row (second row) it means that both amplitudes are equiprobable. Thus, one can interpret a given matrix U as follows. The first row indicates that only a_1 is possible (and it will be updated to this value by the

Gibbs sampler) when the neighboring bottom blob has the amplitude a_1. The second row indicates that either a_1 or a_3 will be selected by the Gibbs sampler with equal probabilities when the bottom blob has the amplitude a_2. The third row indicates that a_3 will be selected by the Gibbs sampler when the bottom blob has the amplitude a_3.

The interaction rule for the horizontal shift

$$U_{[1,0]} = \begin{bmatrix} \infty & \infty & 0 \\ 0 & \infty & 0 \\ 0 & \infty & \infty \end{bmatrix}.$$

These interaction rules do not allow one to use the temperature due to ∞. To make it practical, ∞ was replaced by a large value, such that for the minimal temperature, the corresponding probability is so small that this configuration can never be realized in practice.

THE LUMINANCE OBSERVER

The Luminance observer is based on the distribution of the luminance levels and ignores the geometrical structure of the texture. The performance of the Luminance observer was computed in the framework of Signal Detection Theory (SDT). SDT assumes that the stimulus is encoded into an internal representation and later the decision is made based on the internal representation. In the case of the Luminance observer, the internal representation for each stimulus consists of a luminance histogram. The design of the stimuli simplifies the analysis of the Luminance observer. Instead of analyzing the continuous distribution of luminance histograms, it is sufficient to analyze the distributions of the amplitude indexes $h = (h^1, h^2, h^3)$, where h^k is a number of amplitudes with index k in the texture. Different realizations of textures at the same temperature lead to a distribution of luminance histograms h. In SDT, the performance of the observer depends on the differences in the histogram distributions between the target and distractors and is task dependent.

In the four-alternative-force-choice (4AFC) task (the experimental paradigm adopted here) 4 different textures were presented on each trial: one target (S) and 3 noise (N) textures. One can enumerate spatial locations where stimuli can appear. There are four different configurations: SNNN, NSNN, NNSN, and NNNS. The observer does not know a priori which location target can appear, and computes the likelihood (based on the luminance histogram in our case) for all configurations and selects one having the largest likelihood. Without a loss of generality, one can assume that the target always appears at the first location. Therefore, the Luminance observer correctly identifies the target when the SNNN configuration is selected. The performance of the Luminance observer is computed as the fraction of the trials with a correctly identified target.

It is difficult to analytically compute the form of the distributions and the likelihood function. Therefore, we estimated the performance of the Luminance observer based on 1000 simulated trials at different temperature values. To this end, a pool of 1000 target textures at fixed temperatures and a pool of 3000 noise images were generated. In each trial one target texture and 3 noise images were sampled without replacement from the corresponding pools to estimate the likelihood. The remaining images were used

to estimate the distributions of the target and noise histograms. We employed a kernel density estimation technique with a multivariate Gaussian kernel having an identity covariance matrix. The width of the kernel was chosen such that the performance for $\beta = 0$ was at a chance level. Owing to many assumptions in estimating the density, the performance of our Luminance observer may be substantially suboptimal. Next, the technical details are provided.

Let $s = \{t, n_1, n_2, n_3\}$ denote the stimulus type: the target and noise at locations 2, 3, and 4, correspondingly. Also, let d_s denote the distribution corresponding to the stimulus s. Since distributions for different locations are independent, one can write the likelihood as

$$L_\nu(s_1, s_2, s_3, s_4) = \prod_{\mu=1}^{4} P_\nu(h_{\mu,\nu}|d_{s_\mu}),$$

where $h_{\mu,\nu}$ denotes the luminance histogram observed at location μ and trial ν, s_μ denotes the stimulus at location μ,

$$P_\nu(h_{\mu,\nu}|d_{s_\mu}) = \sum_{n\neq\nu} K_\alpha(h_{\mu,\nu} - h_{s_\mu,n}),$$

where

$$K_\alpha = (2\pi)^{-\frac{N}{2}} e^{-\frac{1}{2}\|\alpha x\|^2}$$

is a Gaussian kernel with width $1/\alpha$, and $N = 3$ is the number of components in vector h. The width of the kernel was chosen large enough ($\alpha = 10^{-5}$) so that the performance at a very high temperature was at a chance level, i.e. there is no overfitting. We did not optimize for α, so it can be substantially suboptimal.

Let $\sigma_\pi(A, B, C, D)$ denote permutations of sequence A, B, C, D enumerated by the index π and $\sigma_{\pi,\mu}(A, B, C, D)$ denote the value in the permuted sequence at location μ. Then the Luminance observer first computes the most likely configuration:

$$C_\nu = \underset{\pi}{argmax}\, L_\nu(\sigma_\pi(t, n_1, n_2, n_3)),$$

and let us assume that the trial is correct when $\sigma_{C_\nu,1}(t, n_1, n_2, n_3) = t$. The performance of the Luminance observer is measured by the fraction of correct trials. Therefore, the Luminance observer is a maximum likelihood Observer based on the luminance histogram measure. It can be interpreted as a Bayesian observer with flat prior. The same is true for other theoretical observers, except that they are based on a different measure.

THE IDEAL OBSERVER

The performance of the Ideal observer was computed the same way as the performance of the Luminance observer, except that a different statistic h was used. More specifically, each texture set was defined by a set of interacting pairs. For each interacting pair, the number of co-appearances of all pairs of amplitudes was computed (3×3 matrix - 9 values). Next, the computed values from all pairs were concatenated to form a single high-dimensional vector h.

THE ORDER OBSERVERS

The performance of the Order observer was based on the order parameter used in statistical mechanics to describe the amount of order present in a physical system. According to one of the definitions, the order parameter reflects the mean conformance between local patches and the overall symmetry of the system, and is, therefore, symmetry specific. Consequently, the order parameter was defined for each texture set separately.

Owing to the finite size of our textures, there is variability in the order parameter across texture realizations, even for the same temperature. This variability plays a role of the internal noise in the framework of Signal Detection Theory. Since it is difficult to obtain an analytical expression for the distribution of an order parameter, we used simulation to estimate the performance of an Order observer. The performance of the Order observer was computed based on 1000 simulated trials. In each trial, the order parameter was computed for the target texture (1) as well as for the distractors (3 noise textures). Next, the Order observer determined what target is based on the maximal value of the order parameter. In cases of a match between the target that was determined by the Order observer and the actual target, the response was considered to be correct. The performance of an Order observer was quantified as the fraction of trials with correct responses.

For all texture sets the order parameter was computed as the mean value of an order parameter field. The specific details regarding computation of the order parameter field for each texture set are presented below.

Texture set 1 To compute the order parameter field, blocks of 8x8 blobs were analyzed at each location. Each block was divided into 4 4x4 non-overlapping squares, and the mean correlation between 2 pairs of vertical squares was considered the value of the order field.

Texture set 2 The symmetry of the Texture set 2 was defined as a repeating structure of 4×4 blocks, where in each block there is a checkerboard-like pattern, where blobs with amplitude a_2 are interleaved with blobs with randomly chosen, a_1 or a_3, amplitudes. Correspondingly, the order field represents this symmetry. It is computed in a few stages. In the first stage, a field b is constructed. The value of the field is set to 1 when the amplitude at the corresponding location is a_2, and is set to -1 otherwise. Then, in each block every second value of field b is changed sign and the mean value in the block is computed. The sign of every value in the block was changed if the value b in the top left corner of the block was negative. In the ordered stimulus every value in the block is equal to 1. In the random stimulus, the expected value is zero. Formally, the order parameter field value (ignoring the translation of large units) for the 4×4 block at location (p, q) was defined as follows:

$$O_{p,q} = \frac{1}{48} \sum_{i,j=0..3} b_{i,j}(b_{\underline{i+1},\underline{j+1}} - b_{\underline{i+1},j} - b_{i,\underline{j+1}}),$$

where

$$b_{i,j} = \begin{cases} 1, & \text{if } A_{p+i,q+j} = 1 \\ -1, & \text{otherwise} \end{cases} , i, j = 0..3$$

and $\underline{k} = k \bmod 4$.

Texture sets 3-5 The symmetry of Texture sets 3-5 is a checkerboard-like pattern, where each check consists of 2×2 blocks of Gaussian blobs. One type of checks consists of blocks of blobs with fixed amplitudes (a_2, a_3, a_1 for Textures 3, 4, and 5, respectively). A second type of checks consists of blocks of blobs, where amplitudes for each block are randomly chosen from the two remaining amplitudes. The order field represents this symmetry. More specifically, the field b is formed as in Texture set 2. The value of the order field is the largest cross-correlation value between field b and the checkerboard pattern represented in the matrix $Q_{k,m}$ below. Formally, the order parameter field value for the 4×4 block at location (p, q) was defined as follows:

$$O_{p,q} = \text{sign}(c_{u^*,v^*}) \min_{\underline{u+v}} \max_{u,v} |c_{u,v}|,$$

where

$$c_{u,v} = \sum_{i,j=0..3} b_{i,j} Q_{\underline{i+u},\underline{j+v}}$$

$$u^*, v^* = \underset{\underline{u+v}}{\text{argmin}} \max_{u,v} |c_{u,v}|,$$

$$Q_{k,m} = \begin{bmatrix} 1 & 1 & -1 & -1 \\ 1 & 1 & -1 & -1 \\ -1 & -1 & 1 & 1 \\ -1 & -1 & 1 & 1 \end{bmatrix}$$

$$b_{i,j} = \begin{cases} 1, & \text{if } A_{p+i \bmod 32, q+j \bmod 32} = a \\ -1, & \text{otherwise} \end{cases}, i, j = 0..3$$

$a = 1, 2, 0$ for Textures 3, 4, and 5, respectively, and $\underline{k} = k \bmod 4$.

Texture set 6 This is a plaid pattern in the ordered state, where the main diagonal amplitudes are either all a_2 or they alternate between a_1 and a_3. Correspondingly, the order parameter field value for the 4×4 block at location (p, q) was defined as follows:

$$O_{p,q} = -\frac{1}{8} \sum_{i,j=0..3} b_{i,j} b_{i+1 \bmod 4, j+1 \bmod 4}.$$

where $b_{i,j} = A_{p+i \bmod 32, q+j \bmod 32} - 1, i, j = 0..3$.

Texture set 7 The symmetry for this set is a checkerboard pattern with interleaving amplitudes a_1 and a_3. The order parameter field value for the 4×4 block at location (p, q) was defined as follows:

$$O_{p,q} = \frac{1}{16} \sum_{i,j=0..3} b_{i,j} b_{i+1,j+1},$$

where $b_{i,j} = A_{p+i \bmod 32, q+j \bmod 32} - 1, i, j = 0..3$.

Texture 8 The order parameter field value for the 4×4 block $b = b_{i,j}, i, j = 0..3$ at location (p, q) was defined as a correlation between the local pattern and the vertical grating patch

$$Q_{k,m} = \begin{bmatrix} 1 & 0 & 1 & 0 \\ 1 & 0 & 1 & 0 \\ 1 & 0 & 1 & 0 \\ 1 & 0 & 1 & 0 \end{bmatrix},$$

where $b_{i,j} = A_{p+i \bmod 32, q+j \bmod 32} - 1, i, j = 0..3$. If all values of $b_{i,j}$ had the same value, the local order parameter value was set to 0.

Texture 9 This is a field with uniform amplitudes a_2. The corresponding order field value is a deviation from a_2. Formally, the order parameter field value for the 4×4 block at location (p, q) was defined as

$$O_{p,q} = \frac{1}{16} \sum_{i,j=0..3} |b_{i,j}|,$$

where $b_{i,j} = A_{p+i \bmod 32, q+j \bmod 32} - 1, i, j = 0..3$.

Texture 10 The order parameter field value for the 4×4 block at location (p, q) was defined as

$$O_{p,q} = \frac{1}{32} \sum_{i,j=0..3} c_{i,j} - 2b_{i,j}b_{i+1,j+1},$$

where

$$c_{i,j} = \begin{cases} 1, & \text{if } b_{i,j} = b_{i+1,j-1} \\ 0, & \text{otherwise,} \end{cases}$$

$b_{i,j} = A_{p+i \bmod 4, q+j \bmod 4} - 1, i, j = 0..3$.

INTEGRATED OBSERVERS

It can be argued that the Order observers we used had adapted to the symmetry of the designed texture set, whereas in Experiments 2 and 3 the human observers did not know to which set a presented target belongs. The same argument may apply for the channel energy model as well. Therefore, here we implemented an Order observer that integrated information from several previously defined Order observers, each designed for a particular texture set (see above), and a Channel energy observer that integrated information from three maps corresponding to three different linear filters, respectively (see the Methods section in the main text). Since the performance for the Order observers for Texture sets 1, 6, and 7 coincide with human performance, we added these observers to the combined order detection model. We also added the Order observer for Texture set 5 in the combined model, since it improved the overall agreement between the Order and human observers. In addition, we combined the responses of 3 Channel energy observers similarly (see the details below). The performances of the combined models are shown in Supplementary Figure 1. The performance of this combined order model approaches the performance of the human

observers in nearly all texture sets. Moreover, it resembles the performance of the combined Channel energy model, thus supporting the conclusions made in the main part of the paper.

THE INTEGRATED CHANNEL ENERGY MODEL

Here the integrated responses for all three Gabor filters were computed for each quadrant, as in the main part of the paper. For each quadrant, we tested the hypothesis that this is an odd ball, i.e. the response computed for this quadrant differs from the responses computed for the other three quadrants. More specifically, the parameters of a Gaussian distribution (mean and variance) were computed based on the responses from the other three quadrants. The likelihood was computed as the probability that the response for the tested quadrant belongs to the same Gaussian distribution. This hypothesis was also tested for all three Gabor filters. The response corresponding to the smallest likelihood among all filters and quadrants (the oddest ball) was selected as the response of the integrated observer.

THE INTEGRATED ORDER OBSERVER MODEL

This observer operated similarly to the combined Channel energy model, except that the hypothesis was based on the order parameter. The hypothesis was tested for the Order observers for texture sets 1, 5, 6, and 7 (see above).

Supplementary Table 1. Interaction rules. Texture sets 1-4

Offset $[p,q]$	$-U_{[p,q]}$
	**Texture set 1**
[4,0]<br>[-4,0]	$\begin{bmatrix} -4.6052 & 0.0100 & -4.6052 \\ -4.6052 & -4.6052 & 0.0100 \\ 0.0100 & -4.6052 & -4.6052 \end{bmatrix}$
[0,4]<br>[0,-4]	$\begin{bmatrix} -4.6052 & -4.6052 & 0.0100 \\ 0.0100 & -4.6052 & -4.6052 \\ -4.6052 & 0.0100 & -4.6052 \end{bmatrix}$
$[0,1]$	$\begin{bmatrix} -2.3955 & -0.6996 & -1.1509 \\ -0.0625 & -0.8078 & -0.6568 \\ -0.2412 & -0.7835 & -0.6524 \end{bmatrix}$
$[1,0]$	$\begin{bmatrix} -0.1892 & -0.9452 & -1.0196 \\ -0.2171 & -0.1965 & -0.0523 \\ -0.4242 & -0.6110 & -0.1211 \end{bmatrix}$
	**Texture set 2**
[1,0]<br>[0,1]	$\begin{bmatrix} -2.3026 & 0.0953 & -2.3026 \\ 0.0953 & -2.3026 & 0.0953 \\ -2.3026 & 0.0953 & -2.3026 \end{bmatrix}$
[4,0] [-4,0]<br>[0,4] [0,-4]	$\begin{bmatrix} 0.0953 & -2.3026 & -2.3026 \\ -2.3026 & 0.0953 & -2.3026 \\ -2.3026 & -2.3026 & 0.0953 \end{bmatrix}$
	**Texture set 3**
[1,0]<br>[0,1]	$\begin{bmatrix} -0.6931 & 0.4055 & -0.6931 \\ 0.4055 & -0.6931 & 0.4055 \\ -0.6931 & 0.4055 & -0.6931 \end{bmatrix}$
[-1,0]<br>[0,-1]	$\begin{bmatrix} 0.4055 & -0.6931 & -0.6931 \\ -0.6931 & 0.4055 & -0.6931 \\ -0.6931 & -0.6931 & 0.4055 \end{bmatrix}$
[2,0] [-2,0]<br>[0,2] [0,-2]	$\begin{bmatrix} 0.4055 & -0.6931 & 0.4055 \\ -0.6931 & 0.4055 & -0.6931 \\ 0.4055 & -0.6931 & 0.4055 \end{bmatrix}$
	**Texture set 4**
[1,0]<br>[0,1]	$\begin{bmatrix} -0.6931 & -0.6931 & 0.4055 \\ -0.6931 & -0.6931 & 0.4055 \\ 0.4055 & 0.4055 & -0.6931 \end{bmatrix}$
[-1,0]<br>[0,-1]	$\begin{bmatrix} 0.4055 & -0.6931 & -0.6931 \\ -0.6931 & 0.4055 & -0.6931 \\ -0.6931 & -0.6931 & 0.4055 \end{bmatrix}$
[2,0] [-2,0]<br>[0,2] [0,-2]	$\begin{bmatrix} 0.4055 & 0.4055 & -0.6931 \\ 0.4055 & 0.4055 & -0.6931 \\ -0.6931 & -0.6931 & 0.4055 \end{bmatrix}$

Supplementary Table 2. Interaction rules. Texture sets 5-10

Offset $[p,q]$	$-U_{[p,q]}$
	**Texture set 5**
[1,0]<br>[0,1]	$\begin{bmatrix} -2.3026 & 0.0953 & 0.0953 \\ 0.0953 & -2.3026 & -2.3026 \\ 0.0953 & -2.3026 & -2.3026 \end{bmatrix}$
[-1,0]<br>[0,-1]	$\begin{bmatrix} 0.0953 & -2.3026 & -2.3026 \\ -2.3026 & 0.0953 & -2.3026 \\ -2.3026 & -2.3026 & 0.0953 \end{bmatrix}$
[2,0] [-2,0]<br>[0,2] [0,-2]	$\begin{bmatrix} 0.0953 & -2.3026 & -2.3026 \\ -2.3026 & 0.0953 & 0.0953 \\ -2.3026 & 0.0953 & 0.0953 \end{bmatrix}$
	**Texture set 6**
[1,1]<br>[1,-1]	$\begin{bmatrix} -0.6931 & -0.6931 & 0.4055 \\ -0.6931 & 0.4055 & -0.6931 \\ 0.4055 & -0.6931 & -0.6931 \end{bmatrix}$
[1,0] [0,1]<br>[-1,0] [0,-1]	$\begin{bmatrix} -0.6931 & 0.4055 & -0.6931 \\ 0.4055 & -0.6931 & 0.4055 \\ -0.6931 & 0.4055 & -0.6931 \end{bmatrix}$
[2,0] [-2,0]<br>[0,2] [0,-2]	$\begin{bmatrix} 0.4055 & -0.6931 & -0.6931 \\ -0.6931 & 0.4055 & -0.6931 \\ -0.6931 & -0.6931 & 0.4055 \end{bmatrix}$
	**Texture set 7**
[1,0] [0,1]<br>[-1,0] [0,-1]	$\begin{bmatrix} -1.6094 & -1.6094 & 0.1823 \\ 0.1823 & -1.6094 & 0.1823 \\ 0.1823 & -1.6094 & -1.6094 \end{bmatrix}$
	**Texture set 8**
[1,0]<br>[-1,0]	$\begin{bmatrix} -1.6094 & -1.6094 & 0.1823 \\ 0.1823 & -1.6094 & 0.1823 \\ 0.1823 & -1.6094 & -1.6094 \end{bmatrix}$
[0,1]<br>[0,-1]	$\begin{bmatrix} 0.1823 & -1.6094 & -1.6094 \\ 0.1823 & -1.6094 & 0.1823 \\ -1.6094 & -1.6094 & 0.1823 \end{bmatrix}$
	**Texture set 9**
[1,0] [0,1]<br>[-1,0] [0,-1]	$\begin{bmatrix} -6.9078 & 0.0010 & -6.9078 \\ -6.9078 & 0.0010 & -6.9078 \\ -6.9078 & 0.0010 & -6.9078 \end{bmatrix}$
	**Texture set 10**
[0,2] [2,0]<br>[0,-2] [-2,0]<br>[1,1]	$\begin{bmatrix} -2.5903 & -2.5903 & 0.0723 \\ -2.5903 & 0.0723 & -2.5903 \\ 0.0723 & -2.5903 & -2.5903 \end{bmatrix}$
[1,0]<br>[0,1][-1,0]<br>[0,-1]	$\begin{bmatrix} -2.5903 & 0.0723 & -2.5903 \\ 0.0723 & -2.5903 & 0.0723 \\ -2.5903 & 0.0723 & -2.5903 \end{bmatrix}$
[1,-1]	$\begin{bmatrix} 0.0723 & -2.5903 & -2.5903 \\ -2.5903 & 0.0723 & -2.5903 \\ -2.5903 & -2.5903 & 0.0723 \end{bmatrix}$

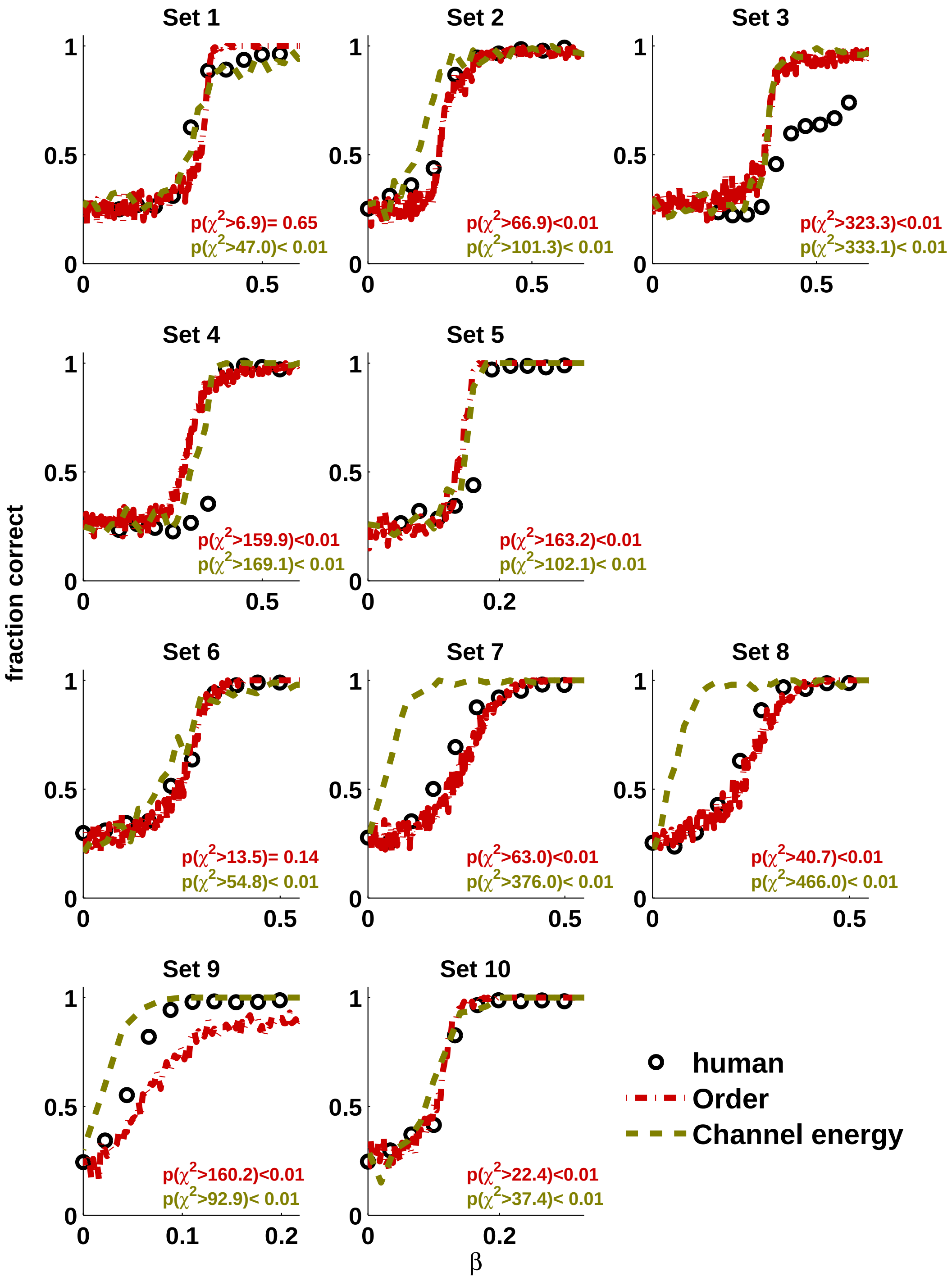

Supplementary Figure 1. Performance of integrated Order (red) and integrated Channel energy (brown) models superimposed on top of human observers. χ^2 statistics for Order (top) and Channel energy (bottom) observers are shown in the inset.
